# *Ask*: a health advocacy program for adolescents with an intellectual disability: a cluster randomised controlled trial

**DOI:** 10.1186/1471-2458-12-750

**Published:** 2012-09-07

**Authors:** Nicholas Lennox, Robert Ware, Suzanne Carrington, Michael O’Callaghan, Gail Williams, Lyn McPherson, Chris Bain

**Affiliations:** 1 Queensland Centre for Intellectual and Developmental Disability, The University of Queensland, Mater Hospital, Raymond Terrace, South Brisbane Qld 4101, Australia; 2 School of Population Health, The University of Queensland, Herston Qld 4006, Australia; 3 School of Learning & Professional Studies, Queensland University of Technology, Victoria Park Road, Kelvin Grove Qld 4059, Australia; 4 Genetics and Population Health Division, Queensland Institute of Medical Research, Brisbane, Queensland, Australia

**Keywords:** Intellectual disability, Health advocacy, Adolescent, School-based intervention, Doctor patient relations, Primary health care, General Practitioner, Health diary, Health check

## Abstract

**Background:**

Adolescents with intellectual disability often have poor health and healthcare. This is partly as a consequence of poor communication and recall difficulties, and the possible loss of specialised paediatric services.

**Methods/Design:**

A cluster randomised trial was conducted with adolescents with intellectual disability to investigate a health intervention package to enhance interactions among adolescents with intellectual disability, their parents/carers, and general practitioners (GPs). The trial took place in Queensland, Australia, between February 2007 and September 2010. The intervention package was designed to improve communication with health professionals and families’ organisation of health information, and to increase clinical activities beneficial to improved health outcomes. It consisted of the Comprehensive Health Assessment Program (CHAP), a one-off health check, and the *Ask Health Diary*, designed for on-going use. Participants were drawn from Special Education Schools and Special Education Units. The education component of the intervention was delivered as part of the school curriculum. Educators were surveyed at baseline and followed-up four months later. Carers were surveyed at baseline and after 26 months. Evidence of health promotion, disease prevention and case-finding activities were extracted from GPs clinical records. Qualitative interviews of educators occurred after completion of the educational component of the intervention and with adolescents and carers after the CHAP.

**Discussion:**

Adolescents with intellectual disability have difficulty obtaining many health services and often find it difficult to become empowered to improve and protect their health. The health intervention package proposed may aid them by augmenting communication, improving documentation of health encounters, and improving access to, and quality of, GP care. Recruitment strategies to consider for future studies in this population include ensuring potential participants can identify themselves with the individuals used in promotional study material, making direct contact with their families at the start of the study, and closely monitoring the implementation of the educational intervention.

**Trial Registration Number:**

ClinicalTrials.gov Identifier: NCT00519311

## Background

Over half a million Australians have an intellectual disability [1]. In this population there are high levels of unrecognised disease, inadequate health screening and promotion, and premature death [2]. We and others have provided some useful information on health checks as a way to improve the health of adults with intellectual disability [3-11]. This project moves on to address another need - the health of adolescents with intellectual disability. The potential for enhancing life-long health and well-being is high in this group, but evidence is lacking.

The few published studies show there are many unrecognised health problems in adolescents with intellectual disability [12,13]. In one 40% of students examined were found to have previously unidentified health issues, with vision and hearing defects being the most prevalent [12]; obesity, orthopaedic problems, dental caries, incomplete immunisations, ENT pathology and skin conditions were also very common [12,13]. Of particular importance is epilepsy, with a prevalence of 20% in this group, as polypharmacy and antiepileptic medication side effects are common and often unrecognised [10,12,14]. During adolescence mental health issues are also important, particularly for those with intellectual disability, whose prevalence of psychiatric disorders is 3–4 times higher than the norm [15-17].

Central to understanding the health of this population are the difficulties they face with communication and recall, which can contribute to the poor reporting of problems [18-20]. As they become more independent from their parents they also face losing the support of their specialist paediatric services and possibly continuity of medical care from their family General Practitioner (GP). This makes it even more important that strategies to minimise the barriers to good healthcare are investigated; these include improved health information systems (records and history taking), health checks, high quality advocacy and education of carers and healthcare providers [18].

A systematic review of the impact of health checks for people with intellectual disability included 38 publications, none of which focussed on the health of adolescents [10]. However there is now substantial trial evidence that adults with intellectual disability who receive health checks experience substantial increases in clinical activities conducive to beneficial health outcomes [9]. GPs perceive a health check in adults as a structured and comprehensive approach to the detection of medical problems as well an aid in overcoming communication problems between the doctor and the person with a disability [21]. Taken together with the high levels of unmet health need among adolescents with intellectual disability, directly assessing the impact of health checks among them makes good sense.

While health checks are a one-off tool designed to increase GPs’ attention to the health needs of individuals with intellectual disability, it is also desirable to ensure longer-term improvement in healthcare advocacy than is achievable using a one-off health check. To this end we have designed and tested among adults a personalised hand-held health record, the *Ask Health Diary*. It contains a record of a client’s personal details, including means of communication, contacts for medical and allied health practitioners, and a medical record of diagnoses, operations, medications, immunisations, allergies, family history of disease and medical consultations [8]. It aims for longer-term enhancement of communication, health records and history taking, and education of carers and healthcare providers. In pilot studies we found that adults with intellectual disability, parents and paid carers who used the diary considered themselves to be better advocates, and that the diary was acceptable for use. They reported improvements in communication, in relationships with their GPs and in organisation of their health information.

The Health Intervention Package is a combination of a health check, the Comprehensive Health Assessment Program (CHAP), and a personalised hand-held health record, the *Ask Health Diary*. The aims of the study are to determine: (1) if adolescents with intellectual disability using this package receive better healthcare (e.g., health screening) and improved health outcomes; (2) if using this package improves health advocacy by adolescents with intellectual disability and their parents/carers in the context of visits to the GP; and (3) if this package is acceptable to adolescents with intellectual disability, their families, their teachers, and their GPs.

The aim of this paper is to describe the pilot study and the trial design of the full randomised controlled trial; to describe which strategies were successful and which were not; and to explore how future projects could be implemented more effectively.

## Methods/Design

### Pilot study

The pilot study examined the implementation of the health intervention package (CHAP health check and *Ask Health Diary*) in adolescents with intellectual disability in one special school [22]. This pilot involved recruitment of 37 students aged 13 years and older with intellectual disability, their parents/carers, the principal and 6 teachers. To introduce the project, we held a presentation at the school; recruitment was assisted by the school principal using telephone and postal communications. In order to actively involve the teachers, the research team’s educational expert met with them and developed curriculum plans including teaching strategies for the *Ask Health Diary*. The diary was distributed to adolescents and used as the basis of a health educational program in six classrooms during the third term of 2004. At the end of the term the Health Intervention Package was taken home to the adolescents’ parents/carers. The family commenced using the *Ask Health Diary*, provided details of the adolescents’ medical history in the first part of the CHAP tool (described below) and organised a health check by their child’s GP. Interviews were conducted with teachers, parents/carers and adolescents before and after receipt of the Health Intervention Package to evaluate its acceptability and efficacy.

A response rate of 72% from the parents/carers was achieved through the support of Education Queensland, the adolescents, their parents/carers, teachers and GPs, and most especially the principal. Results indicated the intervention package was acceptable to all 37 students with intellectual disability, their parents/carers and the six teachers in this school and could be used by adolescents with heterogeneous educational needs. After consultation with Education Queensland, special education teachers, principals and heads of Special Education Schools and Special Education Units, it was clear that to enable use of the new health education material there was a need to fund the schools to relieve the teachers from routine classroom duties. This relief time would allow the teachers to formulate educational strategies for implementation of the *Ask Health Diary* into the teaching plan and enable them to participate in the research interviews. These findings informed the design of the randomised controlled trial as described below.

### Trial design

**Figure 1:**
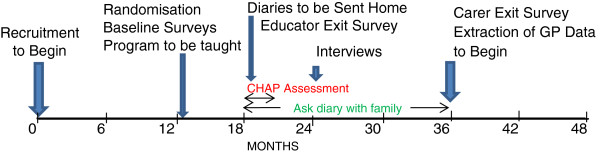
Original Project Timeline.

The study is a parallel group cluster randomised controlled trial testing the usefulness of a health intervention package. The study was conducted among adolescents with intellectual disability in South East Queensland, Australia, between February 2007 and September 2010. The package combines the CHAP health check, the *Ask Health Diary* and the *Ask Project Curriculum Strategy Booklet.* The timeline for the study as proposed is shown in Figure 1.

Ethics approval was granted by both the University of Queensland Behavioural and Social Sciences Ethical Review Committee (Clearance No: 2004000081) and the Queensland Government Department of Education and the Arts (File No: 550/27/424).

### Participants

Participants were drawn from Special Education Schools (SESs) and Special Education Units (SEUs) in Southern Queensland. Children were eligible to participate in the study if they had been assessed by Education Queensland to have an intellectual disability, were aged 10 to 18 years as at 1 January, 2006, and were registered at Education Queensland SESs or SEUs located in Southern Queensland. In Queensland, children who have an intellectual disability may receive their education in a segregated special school for children who have a disability (SES) or in a special education unit or class that is on the campus of a mainstream primary or secondary school (SEU). Students who attend a special school have significant intellectual disabilities and/or multiple disabilities, and usually require specialist teaching and therapy services that support an individualised education program. A small number of these special schools are designated as “High-Support Needs Special Education Schools” and have the staff and specialised facilities required for children with profound disabilities. Students who attend a special education unit in a primary or secondary school may have a range of disabilities, and usually access the mainstream curriculum and receive specialist teaching and therapy services. The choice of placement options for students with disabilities can be influenced by a range of factors, such as parent choice and education options available in the local area.

### Recruitment

Under the terms of the ethical clearance provided by Education Queensland it was necessary to obtain consent from the relevant principals/heads before research could be conducted, but before this could be obtained, the following process was undertaken:

• *Central executive level contact*. Although participant recruitment did not occur at this level, the purpose of this executive level contact was to: (a) secure direct support from a Senior Executive for the study (as indicated by a written letter of support); (b) seek advice on appropriate strategies to recruit SESs and SEUs within Southern Queensland; and (c) access the names and contact details of schools. Support was obtained for the study to be conducted in State schools. Catholic and some other independent schools were also approached, but their recruitment was considered too difficult in the time frame.

• *Regional and district level contact*. The chief investigator contacted the district directors of all schools in Southern Queensland to: (a) provide information about the study; (b) seek district-level support for the study; and (c) obtain the names and contact details for Principal Education Officers (Student Services) in each district.

• *Principal Education Officers – Student Services (PEOs).* The research officers responsible for recruitment then contacted by telephone all of the PEOs in Southern Queensland, which was followed up by an emailed information package. The purpose of this contact was to: (a) provide information about the study; (b) seek PEO support for the study; and (c) identify opportunities to discuss the study with principals and Heads of Special Education Services of the SESs and SEUs in each PEO’s jurisdiction.

• *Principals and Heads of Special Education Services (HOSES).* The project manager and/or the research officer responsible for recruitment then contacted Principals of the SESs and HOSES of the SEUs by telephone to introduce the study. The purpose of initial telephone contact was to (a) determine the eligibility of each SES/SEU to participate in the study; and (b) identify opportunities for speaking to parents/carers about the study. The recruitment officer then arranged to visit all interested schools to describe the project to teachers. The *Ask Project Curriculum Strategy Booklet* was made available on the web for staff to peruse.

If schools agreed to participate, information sheets and consent forms were provided for distribution to parents/carers of possible participants via school newsletters or communication books. As some of the parents of these adolescents also had disability and literacy problems, a DVD was produced to describe the program and was distributed to families.

The name and contact details of the adolescent’s usual GP was provided by the parent/carer of each participant at baseline. A brief introductory letter describing study objectives, research design and data collection process, and the type and level of participation required was sent to each nominated GP, followed by a phone call from research staff. A consent form and detailed information sheet was faxed to doctors; this included information on how the GP could claim a government funded fee for providing a comprehensive health check. On completion of GP recruitment a thank you letter was sent to consenting GPs advising them of the participants who had nominated them as their usual GP.

### Sample size

We estimated there would be 2,403 adolescents aged 13 to 18 years and registered at SES or SEU, based on Education Department data from the year prior to study commencement. Approximately 80% of these adolescents would have intellectual disability; meaning approximately 1900 students would be eligible for the study. We estimated that around 60% of schools would choose to participate in the study, and that within each school 95% of eligible individuals would participate. Consequently we expected to have a total sample size of around 1,000 adolescents (80% x 60% x 95% of 2403). We estimated participants would be grouped into around 50 clusters (assuming that an average of 20 (SD = 7.5) adolescents with intellectual disability would attend any one school). Thus we expected that each of the intervention and usual care groups would comprise 500 students from 25 schools.

One of the primary outcomes of interest is detection of vision or hearing impairment, which previous studies have shown has a current rate of around 10% in this population [12,13]. With an assumed intra-class correlation coefficient (ICC) of 0.03, the design effect for this analysis is 1.57, without consideration of matching. On this basis, the study would have 90% power to detect an increase in the detection of vision impairment to 19% due to the intervention, at the 5% level of significance. For a condition with a current impairment rate of 20%, the study will have 90% power to detect an improvement to around 30% through the intervention. The matched-pairs design will have greater power, depending on the within-pair correlations, so the above estimates are conservative. The assumption of a maximum 0.03 for the ICC is plausible, based on a reported ICC (0.01) in an adult population [7]. For analyses of major subgroups, e.g. examining increases within adolescents with Down syndrome, an estimated 25% of the sample distributed across clusters, the study would have 80% power to detect increases of 10% to 24%, or 20% to 37%.

### Randomisation

Allocation in this study was by cluster randomisation. The unit of randomisation was the school. Individual randomisation was inappropriate for this study as the teaching had to be done in the open classroom, even if lessons were individualised for each student. Consequently it was essential all students in a particular school were allocated to receive the same treatment (either the intervention or usual care).

Randomisation occurred after schools were stratified by type (Special Education Schools/Special Education Units/High Support Needs Schools) and location (Major City/Inner Regional/Outer Regional). Location was categorised according to the Accessibility/Remoteness Index of Australia classification [23]. Within each stratum, schools were ranked according to the expected number of participating adolescents. Using the ranked list within each stratum, schools were formed into pairs. A list of computer-generated random numbers was used to allocate either the intervention or usual care treatment to the first of each pair; the second in each pair was allocated the alternative treatment.

### Intervention

The intervention school adolescents received the Health Intervention Package, consisting of: an *Ask Health Diary*, an *Ask Project Curriculum Strategy Booklet* and a CHAP health check for use by the adolescent, their parents/carers and GPs. The usual care group received their usual education and medical care. The *Ask Health Diary* component involved the adolescent and the teacher in the classroom, and the adolescent, parent/carer and GP in the surgery. In schools the *Ask Health Diary* was used as a curriculum resource for teaching.

The *Ask Health Diary* has four principal sections: personal details about the adolescent; advocacy tips; guidance for the GP; and health records. By using the first section called “All About Me”, the student learned how to fill in practical details, such as names and addresses of all the health professionals involved in the person’s health care. The student could also role-play attending a GP consultation.

From the second section, “Health Advocacy Tips”, the teacher could use lessons to teach the adolescent tips for general good health. For example, they could be taught practical knowledge about the doctor’s surgery, and skills in organisation for before, during and after going to the doctor. Picture pages assist in communicating symptoms, and diary pages permit keeping track of potentially problematic areas such as menstruation, bowel, bladder, epilepsy and other relevant issues.

The third section, “For the Doctor” provided the GP with information about significant, often unrecognised or poorly managed health problems that adolescents with intellectual disability have, such as inadequate immunisation or constipation, as well as practical tips for doctors and clinic staff, and a syndrome co-morbidity check list.

The fourth section “Medical Records” includes a list of commonly missed problems to be checked, and areas for new findings (e.g. hearing and vision impairments) and actions taken or planned. Additional pages allow recording of diagnoses, operations, medications, immunisations and other histories of relevance.

The *Ask Project Curriculum Strategy Booklet* developed in the pilot provided teaching ideas, strategies and resources to assist teachers with their planning.

The CHAP component of the package involved interactions between the adolescent, the parent/carer and the GP. The adolescent and their parent/carer filled out the first part of the CHAP booklet. This collected a broad history of the adolescent’s health and provided the GP with information that may have needed further investigation. The second part of the booklet was for the GP, who was asked to review the history provided, and then to perform a targeted health examination and record findings. The booklet also acted as an educational tool for GPs, as it gave syndrome-specific physical and mental health problems and prompted the GP in relation to unrecognised or poorly managed disease and unmet preventative health needs.

Based on the pilot study teacher interviews, the *Ask Health Diary* was scheduled to be incorporated into the curriculum throughout the first two terms in the intervention group schools. At the end of Term 2 the diaries were to be sent home and the CHAP booklets sent to the parents/carers, who were to be asked to complete the history gathering section of the CHAP and make an appointment to see the GP during Term 3. Teachers were asked to recommend to carers that the *Ask Health Diary* be used for subsequent on-going contact with the GP and other health service providers. Research staff contacted parents/carers in July 2008 to ensure they had received a diary from the school. A diary was immediately forwarded directly to participants who reported they had not previously received one.

### Data collection

Baseline surveys were sent to educators in both the control and usual care groups. Five months after baseline teachers who taught the program were sent an exit survey. For all adolescents, carer baseline and exit surveys were sent and GP data collected. Carer exit surveys, sent 26 months after baseline, were the same for both intervention and usual care groups, with the addition of CHAP and diary-specific items regarding their usability and perceived benefits for the intervention group. After each GP visit, the CHAP health check booklets were returned to the research team by the carers of adolescents in the intervention group. Evidence of health promotion, disease prevention and case-finding activities were extracted from GPs clinical records 28 months after baseline and after all participants would have had an *Ask Health Diary* for at least 14 months. Qualitative interviews of educators and adolescents occurred after completion of the educational component of the intervention.

#### Baseline surveys

Baseline surveys were sent to educators and carers at the commencement of the intervention. Educator surveys aimed to gather data on age, sex, position and experience of the educator; communication methods of students; school record keeping of students’ current medical information, previous teaching of health related matters and the educator’s concept of advocacy. Carer surveys asked about the age and sex of the adolescent; the aetiology of their intellectual disability; their abilities; and for an assessment of their health. Information on household demographics, their medical record keeping, communication with the school and their reasons for and difficulties involved in their participation in the project were also requested. A measure of the adolescent’s health advocacy skills and the Short Form of the Developmental Behaviour Checklist (DBC-P24) [24] was also included. Schools were contacted to provide participant’s IQ and Adaptive Behaviour scores.

The pilot study [25] found that some parents have low literacy skills and for this reason responding to a questionnaire might be problematic; so telephone interviews were offered when both the baseline and exit surveys were distributed and again on follow-up. All carers who had not responded received two telephone reminder calls.

#### Qualitative interviews

Semi-structured interviews were conducted with a small convenience sample drawn from three groups of individuals from the intervention group: adolescents, their parents/carers and teachers. Teachers were invited to interview in July 2007, which was as soon as practicable after teaching the program. Questions were designed to elicit information around the following themes: how and how often the diary was used in the classroom; difficulty or ease of use in the classroom; and suggestions for improvement for use as a teaching tool. Feedback about the perceived benefits of the diary was also requested.

Adolescents and their carers recommended by these schools were interviewed separately in June 2008 after they had completed the CHAP review. The adolescents were interviewed face-to-face at school. Conducting interviews with young adults who have an intellectual disability was a challenge. The researcher who conducted these interviews was an experienced special education teacher and researcher, who had worked extensively with adolescents with intellectual disability. The semi-structured interviews took place at each school in a quiet location where the students were comfortable. The interview schedule was designed to find out how students used the diary and how it was helpful to them. Care was taken to develop rapport with each student and visual prompts, such as displaying a copy of the *Ask Health Diary* to remind the students what the questions were about, were used.

For the parents/carers, questions were designed to elicit information around the following themes: styles of communication; barriers and opportunities for communication; methods of working with GPs; styles of advocacy and determination of decision-making processes during doctor visits, particularly with regard to the CHAP. Interviews with parents/carers were conducted by telephone. We asked for feedback on how the intervention package assisted, or otherwise, participants’ interactions with their GPs; on how advocacy skills were improved; on the tool used in the health review; and on how the intervention package might be improved or altered.

#### Exit surveys

The results of the face-to-face teacher interviews conducted after teaching the program were used to inform the teachers’ post-intervention survey which was distributed at the end of Term 3. All teachers reported to have taught the program were asked about their teaching method, the use of the diary, any problems they faced, and for suggestions to improve the diary. Items were designed to allow them to rate their effectiveness in teaching this material, report any perceived gains for adolescents and their possible future use of the diary. They were also asked to indicate the effect of the program on their expectations of students.

All parents/carers received a post-intervention postal survey at least 12 months after receiving the *Ask Health Diary* and over 16 months after the median CHAP health check date. They were asked about the use of the diary and the CHAP. Items from the baseline on household demographics, the DBC-P24, the measure of health advocacy and the assessment of the adolescent’s health were repeated in this exit survey. Items on residential mobility and their lifestyle and living situation were also included.

#### Health outcome data

In September 2009 we began the process of collecting data from the participants’ usual GPs. A letter was sent to each practice manager advising them of our wish to come and collect the data. We requested copies of all medical records, including correspondence and results of investigations from January 2006 to date of contact with the practice. Approximately one week later this was followed up with a phone call to the practice manager and a date set for data collection. For security reasons, the data were collected from practices by project staff rather than being posted or faxed. Most practice records were computerised and practice managers or doctors produced a print-out of all the data which we collected. In non-computerised practices, photocopies of files were obtained if possible.

Data on medical disorders recorded, past and family histories, allergies, smoking and alcohol consumption, immunisations, current medications, health screening, traumatic injuries, hospitalisations, weight management and the use of the diary was extracted from the notes provided by the GPs. The diagnosed medical disorder was then coded according to both the International Statistical Classification of Diseases and Related Health Conditions (ICD-10) and the International Classification of Primary Care (ICPC-2-R) systems. If there was no diagnosis made, the presenting symptoms were coded. New diagnoses were then identified. A medical professional with extensive experience in the field of intellectual disability checked all ICD codes and new diagnoses. Challenging behaviour is a major issue in this population and much data was available in the GP records collected. All reported challenging behaviours as defined by Emerson [26] were recorded. The data extraction process was refined from study methods successfully used in other projects conducted by the research team [6,7]. Although we attempted to mask data extractors to a participant’s treatment status, in practice this was not wholly successful as in many cases the GP had recorded in the notes that his patient had attended his surgery for a CHAP. In a previous research project where the same data extraction tool was used, no difference was found when comparable patterns of health actions were recorded for individuals who received the CHAP regardless of whether or not the extractor was masked [6].

### Analysis

#### Qualitative

All the interviews which had been recorded verbatim were transcribed and organised using the software NVivo (version 8.3). Demographic data from Baseline surveys were converted from Access to Unicode Text (*.txt) and imported into NVivo as a casebook. The responses to the open ended questions in the Teacher Exit Survey were exported from the Access database, converted to Word Documents, imported into NVivo and coded using the Auto Code function. This process ensured that an integration of the data sets could be achieved [27]. We generated categories and sub-categories from the segments of the data, usually sentences and paragraphs; constantly checking the emergent understandings and clarifying these with the research team [28]. The responses to the open-ended questions in the carer exit survey will be analysed in the same manner.

#### Quantitative

Percentages of detection of health screenings and health promotion activities initiated, and responses to postal questionnaires will be compared between the intervention and the usual care group using data extracted from medical records. The individual will be the unit of analysis. For continuous outcome variables, a mixed-effects linear regression model will be used to compare the outcomes between comparison groups, with allowance made for cluster and matching effects. For categorical outcomes, a mixed-effects logistic regression model will be used. Sub-group analysis of adolescents at particular risk, such as those with Down syndrome will also be performed.

The primary analyses will be based on the ‘intention-to-treat’ (ITT) principle; all individuals with evaluable data for the outcome under investigation will be analyzed in the group they were randomized to, regardless of treatment received. Additionally, ‘per protocol’ (PP) analyses will be conducted where there have been large deviations from the planned intervention; all individuals with evaluable data for the outcome under investigation will be analyzed according to the treatment received. Any such analyses will be clearly labeled as such and cautiously interpreted as perhaps indicating the maximum theoretical potential of these programs. The PP analyses will consist of three comparison groups according to the timing of the treatment received. The three groups are ‘intervention early’, ‘intervention late’, and ‘no intervention’. The ‘intervention early’ group will consist of individuals whose teachers reported they received their *Ask Health Diary* at the time they were scheduled to, or within 3 months of the time they were scheduled to, and parents/carers confirmed this had been received. The ‘intervention late’ group will consist of individuals who teachers reported they received their *Ask Health Diary* at least three months later than they were scheduled to and those who, when their parents/carers were contacted in July 2008, reported they had not received a diary. The ‘usual care’ group will consist of individuals randomized not to receive an intervention. The separate effects of the CHAP health check, the *Ask Health Diary* and the *Ask Project Curriculum Strategy Booklet* will be explored by comparing outcomes for individuals according to the number, and timing, of components of the health intervention package they received.

## Results

### Recruitment

**Figure 2:**
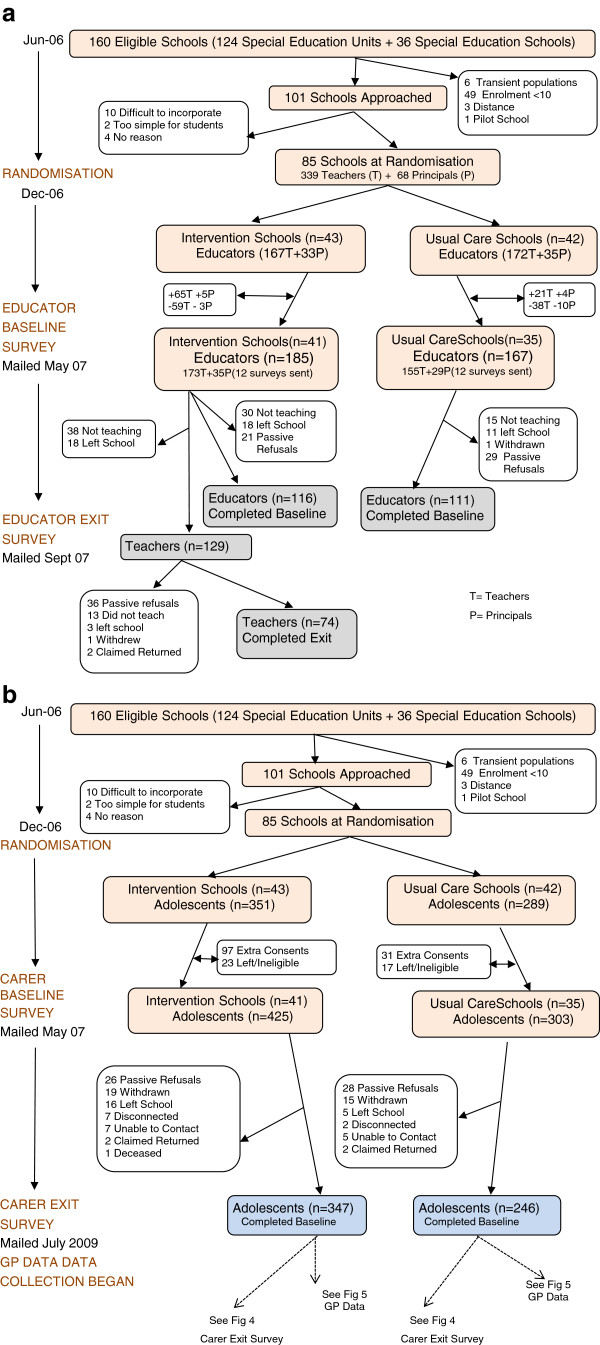
a Participant Flow – Educators, b Participant Flow – Adolescents and Carers.

Data were provided by Education Queensland as to the number of students in the 160 SESs and SEUs that met the inclusion criteria. Of the 160 schools, 59 were excluded as 49 had enrolments of less than 10, one had participated in the pilot project, six had student populations that were transient in nature and three schools were considered too remote. The recruitment officer contacted all eligible schools and offered to do a presentation for teachers. Of the 101 schools approached, 16 schools declined. Of these 10 considered it too difficult to incorporate the project into what was often an already over-crowed curriculum, two special education units thought it too simple for their students and four gave no reason (See Figure 2a).

### Intervention

The program was scheduled to be taught in schools in the first term of 2007, but changes in the way teachers were allocated to schools at the beginning of that year meant that in first term there were major staff changes and, as a result, after randomisation but before we had sent out baseline surveys, we saw nine schools withdraw from the study. All schools reported difficulty in obtaining student consent, but more usual care schools than intervention schools withdrew from the study (See Figures 2a and 2b). New teachers in special education units randomised to the intervention group considered it appropriate to teach the program to incoming Grade 8 students which resulted in the inclusion of 12 year old adolescents in the study. Because of the organisational structure of special schools, some even younger students were also included which led to the revision of our age eligibility criteria to 10–18.

### Data collection

#### Baseline surveys

The staff changes after randomisation resulted in 185 educators from the 41 intervention schools and 167 from the 35 usual care schools participating at baseline (See Figure 2a). At baseline there were a total of 728 adolescent participants in the study, 425 in the intervention group and 303 receiving usual care (See Figure 2b). After intensive follow-up we achieved a response rate of 64.5% (n = 352) for educator and 81.5% (n = 728) for carer surveys. Information was obtained directly from schools on intellectual functioning for 203 of the adolescents.

#### Qualitative interviews

Individual interviews were conducted with nine special school teachers and four special education unit teachers. A group interview was conducted with three teachers from one special education unit. Sixteen adolescents and their carers were interviewed with only eight carers reporting that they had received a diary from school. All participants interviewed had done the CHAP as requested.

#### Exit surveys

Seventy-four (57.4%) of the 129 teacher exit surveys mailed were returned for analysis (See Figure 2a). Only 26 teachers reported that they began teaching the project in Term 1; 34 did not do so until Term 2; and others began even later in the year. Teachers also differed in the number of hours per week and the number of weeks taught. 47 teachers taught for one semester only, 23 taught over 2 semesters and 4 taught over 3 semesters. On average teachers taught for 11 weeks (SD = 7) and for 57 minutes per week (SD = 28).

**Figure 3:**
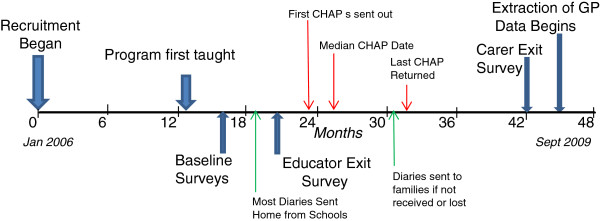
Actual Project Timeline.

Most diaries were not sent home from schools until June/July 2007, but others were not sent until third or in at least two cases fourth term. CHAP booklets were sent to carers of the intervention participants after all schools reported that the diaries had been sent home so that they might take their child to their doctor for the health check in November 2007. These delays required a revision of the original project timeline (Figure 1). Figure 3 shows the actual timeline.

**Figure 4:**
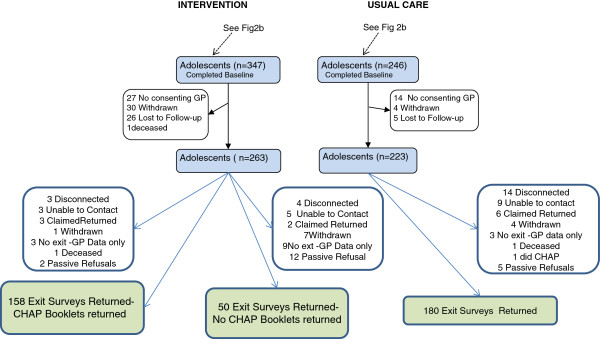
Participant Flow – Carer Exit Survey.

Between Baseline and the Exit many participants were lost to follow up and 41 had no consenting GP so could not continue in the study. Of the 347 adolescents from the intervention group who completed the baseline survey, only 176 returned their CHAP booklets. Of the 486 carer exit surveys sent out in July 2009, 388 (79.8%) were returned (See Figure 4).

##### Health outcome data

**Figure 5:**
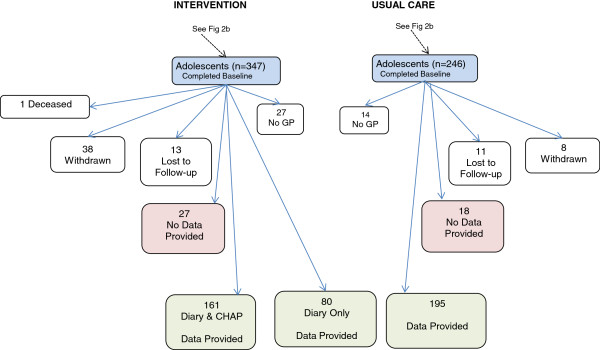
Participant Flow – GP Data Collection.

GP records were collected for 436 of the participants (See Figure 5). Depending on the time of collection and whether or not all GPs the adolescent had attended during the period had consented to participate in the study, data is available for each participant for a period up to 54 months between January 2006 and June 2010. Data was obtained for 195 of the usual care group, 161 of the intervention group who had completed both the educational and medical components of the intervention and 80 who had the educational intervention only, i.e. they did not do the CHAP.

## Discussion

We describe the methodology employed that enabled us to perform a pilot and randomised controlled trial involving a hard-to-reach population in a complex naturalistic setting. Although we were required to modify the original protocol due to the nature of this population and the research environment, we maintained the integrity of the study. While our aim was to recruit 1000 participants, the sample size of 728 was still large and good response rates were obtained from both carers and GPs.

Our research strategy was based on our experience of trials in this population and the known barriers to conducting research in adults with intellectual disability. We used materials developed or informed by the previous randomised controlled trials in adults, such as CHAP booklets that have been demonstrated to be acceptable to people with intellectual disability and their families. We believe our success was also based on our awareness that decision-making in government departments and schools can be a political process [29]. Our approach involved active communication and the development of relationships with all levels of the education system. We met with several tiers of governance in the Queensland Education Department to inform them and gain their support before accessing the schools and teachers. To maximise their understanding of the project, we provided direct contact and accessible written information. At all levels we attempted to convince staff that this project should be included in an already overcrowded curriculum. Prevention programs inevitably place extra burdens on school and student respondents [30], so in order to encourage participation, schools were reimbursed for staff time. We persisted with reminders and consistent follow-up with school principals, senior, and classroom based, teaching staff. This approach appears to have diminished the known barriers and increased our ability to implement the research protocol.

There were also approaches we took which did not work as well as anticipated. We found a successful pilot project did not provide sufficient breadth of understanding of the usual situation in the school system to adequately inform the larger project. The principal in our pilot special school was enthusiastic, energetic and organised, characteristics found to aid implementation of school-based prevention programs [31]. These qualities were not always present in liaison staff from other schools. Furthermore, pilot study teacher interviews suggested the project should be conducted in Term 1, but in hindsight other schools may have found it easier to incorporate it into the curriculum mid-year. If the pilot had been conducted in a special-education unit as well as this special school, we may have been better prepared for the recruitment and implementation difficulties that followed. We developed a DVD designed to assist those parents who might have trouble understanding the written material. This approach appeared to assist in recruitment in the special school setting, but anecdotal reports from special education units indicated that the interviews with students of special schools on the DVD were not viewed positively by students with lower support needs who usually attend special education units in mainstream schools. If we use this approach in the future, we would ensure that participants can identify themselves with the individuals used in the promotional materials.

We were attracted to school-based prevention research for adolescents as we could target a large proportion of the population [32], but as others have reported, based on research with non-disabled adolescents, these studies are not without their problems [29,30,32]. We believe some of the difficulties we experienced in gaining consent from parents and other carers may have been because research staff had no direct contact with families at this early stage of the trial. Fletcher and Hunter [33] found that consent rates were low in classrooms that had teachers who were either invested minimally in the project or were new to schools. Many teachers, new to our schools at the beginning of the year, had been asked to teach the project at short notice and so may not have been as enthusiastic or prepared as their counterparts who had agreed to do so at the end of the previous year. In hindsight we would recruit in the first six months of the school year and introduce the *Ask Health Diary* and the CHAP in the second half of the year.

A further difficulty we experienced was the lack of control the researchers had over implementation. We provided the teacher with an *Ask Project Curriculum Strategy Booklet* which they used only as a guide, leading to substantial variation in the way the program was taught. This flexibility is appropriate in school settings because the teacher will need to adjust their approach based on their intimate understanding of the characteristic of their students.

We also found the diary was not always sent home at the appropriate time as 44% of the parents/carers of participants were not aware of the diary when contacted by research staff twelve months after it was scheduled to have been sent home from school. Anecdotal evidence suggests there was also less than optimal teacher-parent communication as to the purpose of the diary and the CHAP. Families often did not take the adolescent for the health review. The Carer exit survey may reveal other reasons for this, but if we were to repeat this study we would be in more constant contact with the teachers to ensure they complied with the protocol.

People with intellectual disability throughout the world are too often left behind in the progress that is made in improving the health of other citizens [34]. Adolescents with intellectual disability face enormous difficulties in obtaining even the most basic health care. There are few avenues for them to become empowered and to improve and protect their own health. There are also too few GPs who have received adequate training in this area, and the health care system offers few incentives to ensure appropriate care for people with special needs [18] Because of these factors, individuals with intellectual disability suffer health disparities, which must be addressed [18,35-37]. This project seeks to achieve health gains with an intervention, which at its core empowers adolescents with intellectual disability (and their families) as they move towards the increased autonomy of adulthood. It is focused at a time when they are moving away from the relative protection of special education providers and targeted paediatric health services to limited and, possibly inadequately trained, generic health-care providers. This project seeks to demonstrate that the health of this population can be improved through: augmentation of communication and good advocacy; improved documentation of health problems; and improving access and quality of GP care. With an individualised health check and an individualised health advocacy diary for improving health care skills, the adolescent may gain a solid basis for, and an expectation of, good health care. These steps may not alleviate the all of problems but should pave the way to a noticeable improvement in both the processes and outcomes in health care.

## Competing interests

Uniquest, a corporate arm of the University of Queensland licenses the CHAP for use. The royalties from this license are shared equally between the University, QCIDD and the first author. The other authors declare that they have no competing interests.

## Authors’ contributions

NL, SC, MOC, GW and CB obtained funding for the research. All authors contributed to the conceptual design of the study and provided intellectual input into the design of this paper. All authors approved the final version of this manuscript.

## Pre-publication history

The pre-publication history for this paper can be accessed here:

http://www.biomedcentral.com/1471-2458/12/750/prepub
